# Introducing a Novel Personalized Microbiome-Based Treatment for Inflammatory Bowel Disease: Results from NostraBiome’s Internal Validation Study

**DOI:** 10.3390/biomedicines13040795

**Published:** 2025-03-26

**Authors:** Adrian Goldiș, Radu Dragomir, Marina Adriana Mercioni, Christian Goldiș, Diana Sirca, Ileana Enatescu, Oana Belei

**Affiliations:** 1 Department of Gastroenterology and Hepatology, “Victor Babeș” University of Medicine and Pharmacy, 300041 Timișoara, Romania; goldis.eugen@umft.ro; 2 Department of Obstetrics and Gynecology, “Victor Babeș” University of Medicine and Pharmacy, 300041 Timișoara, Romania; 3 Faculty of Medicine, “Victor Babeș” University of Medicine and Pharmacy, 300041 Timișoara, Romania; marina.mercioni@student.umft.ro (M.A.M.); christian.goldis@student.umft.ro (C.G.); diana-cristiana.sirca@student.umft.ro (D.S.); 4 Applied Electronics Department, Faculty of Electronics, Telecommunications and Information Technologies, Politehnica University Timișoara, 300223 Timișoara, Romania; 5 Twelfth Department, Neonatology Clinic, “Victor Babes” University of Medicine and Pharmacy, 300041 Timisoara, Romania; enatescu.ileana@umft.ro; 6 First Pediatric Clinic, Disturbances of Growth and Development on Children Research Center, “Victor Babeș” University of Medicine and Pharmacy, 300041 Timișoara, Romania; belei.oana@umft.ro; 7 First Pediatric Clinic, “Victor Babeș” University of Medicine and Pharmacy, 300041 Timișoara, Romania

**Keywords:** inflammatory bowel disease, microbiome, gut dysbiosis, personalized medicine, probiotics, biologic therapy, fecal calprotectin, inflammatory markers, AI-driven therapy

## Abstract

**Background/Objectives:** Inflammatory bowel disease (IBD), encompassing ulcerative colitis and Crohn’s disease, is characterized by chronic gut inflammation driven by microbial dysbiosis and immune dysfunction. Current therapies primarily involve anti-inflammatory and immunomodulatory strategies; however, many patients experience an inadequate response or a gradual loss of efficacy over time. This study evaluates the clinical efficacy of personalized microbiome modulation (PMM)—an AI-driven intervention designed to restore microbial balance and improve key treatment outcomes such as symptom control and remission rates. **Methods**: This was a single-arm, open-label validation trial involving 27 patients with moderate-to-severe IBD who had experienced prior treatment failure. Participants underwent three months of PMM, which included personalized dietary modifications, targeted probiotic supplementation, and antimicrobial interventions based on gut microbiome sequencing. Primary outcomes included stool frequency and consistency as well as inflammatory markers (C-reactive protein and fecal calprotectin), while secondary outcomes assessed nutritional status, metabolic function, and quality of life. Statistical analyses included paired t-tests and repeated measures ANOVA to determine significant changes over time. **Results**: PMM led to significant clinical improvements, including a 58% reduction in stool frequency (*p* < 0.001) and improved stool consistency. CRP and fecal calprotectin levels decreased markedly (*p* < 0.001), suggesting reduced systemic inflammation. Additionally, iron, vitamin B12, and vitamin D deficiencies improved (*p* < 0.001), alongside weight gain and increased energy levels. Notably, patients on anti-TNF biologics showed enhanced response rates, suggesting potential synergistic effects between microbiome modulation and biologic therapy. **Conclusions**: This study highlights PMM as a promising adjunctive therapy for IBD, demonstrating benefits across clinical, inflammatory, and metabolic parameters. While findings support the role of microbiome-targeted interventions in disease management, larger randomized controlled trials are required to confirm the long-term efficacy and applicability in broader patient populations.

## 1. Introduction

Inflammatory bowel disease (IBD), comprising ulcerative colitis (UC) and Crohn’s disease (CD), is a chronic, immune-mediated condition characterized by persistent gastrointestinal inflammation, resulting in severe morbidity and impaired quality of life [1]. Despite advances in medical therapy, including immunosuppressants, biologics, and small-molecule inhibitors, a significant subset of patients experience partial or no response, requiring frequent treatment escalation or surgical interventions [2]. Given this therapeutic variability, the multifactorial nature of IBD, involving genetic predisposition, immune dysregulation, environmental triggers, and gut microbiome alterations, has necessitated the exploration of novel therapeutic strategies beyond traditional immune modulation [3].

In this regard, emerging evidence suggests that gut dysbiosis, or microbial imbalance, plays a central role in IBD pathogenesis. Several studies have reported reduced microbial diversity, increased pro-inflammatory bacterial species (e.g., *Escherichia coli*, *Fusobacterium nucleatum*), and decreased beneficial commensals (e.g., *Faecalibacterium prausnitzii*, *Akkermansia muciniphila*) in IBD patients [4,5,6]. These alterations contribute to intestinal barrier dysfunction, aberrant immune activation, and chronic inflammation, thereby perpetuating disease activity. However, traditional treatments fail to directly address microbiome composition, leading to inconsistent long-term remission rates and persistent disease symptoms [7].

To address this limitation, NostraBiome has developed an AI-driven, hyper-personalized microbiome modulation (PMM) approach that tailors interventions based on an individual’s gut microbial profile. Artificial intelligence (AI) has emerged as a powerful tool in microbiome research, facilitating the identification of microbial dysbiosis patterns and optimizing treatment strategies in IBD [8]. This method integrates advanced microbiome sequencing (16S rRNA), machine learning algorithms, and clinical profiling to generate customized dietary, probiotic, and antimicrobial recommendations to restore microbial balance [9].

Complementing this perspective, recent research highlights the crucial role of gut microbiota alterations in IBD progression, influencing intestinal barrier integrity and immune responses [7]. As conventional treatments do not directly address dysbiosis, microbiome-targeted interventions are emerging as potential therapeutic strategies. Various approaches, including probiotics, prebiotics, and fecal microbiota transplantation, have shown promise in modulating gut microbial composition and improving clinical outcomes [3].

Building on this context, the present study explores the impact of a personalized microbiome-based intervention on IBD management. The primary objective was to assess changes in stool frequency and consistency and inflammatory markers, such as C-reactive protein (CRP) and fecal calprotectin—widely recognized indicators of disease activity [10]. Secondary outcomes also included nutritional status, quality of life, bloating, and fatigue given the high prevalence of dietary deficiencies in IBD due to chronic inflammation and microbial imbalances [11].

Understanding the potential of microbiome modulation as an adjunct to standard IBD therapy is essential, particularly in optimizing treatment response and long-term disease remission. While preliminary findings suggest beneficial effects, further randomized controlled trials (RCTs) are necessary to confirm these results and explore their applicability in broader patient populations, including pediatric cases [12,13].

## 2. Materials and Methods

### 2.1. Study Protocol, Data Collection, and Follow-Up

This single-arm, open-label internal validation trial investigates the efficacy of AI-driven PMM in individuals with IBD, specifically UC and CD. The primary goal is to assess improvements in stool frequency, consistency, and inflammatory markers, while secondary objectives focus on evaluating nutritional status, quality of life, bloating, and fatigue.

A total of 27 participants, aged 26–55, with moderate-to-severe IBD and a history of treatment failure were included. Over three months, participants underwent regular evaluations, including microbiome sequencing, inflammatory marker analysis (CRP, fecal calprotectin), and clinical symptom tracking.

Data collection encompassed self-reported stool diaries, 16S rRNA sequencing for microbiome profiling, blood tests for inflammation and nutritional deficiencies, and quality-of-life assessments. Statistical analyses, including paired t-tests and repeated measures ANOVA, were used to determine significant changes over time.

Participants followed a structured three-month monitoring protocol, with assessments at baseline (Day 0), Month 1, Month 2, and Month 3. These evaluations tracked bowel movement frequency, stool consistency (Bristol Stool Scale), and blood in stool through self-reports and clinical verification. Additionally, bloating and gas levels were recorded based on patient-reported discomfort scores.

Biochemical and nutritional parameters were assessed to evaluate both systemic inflammation and malabsorption-related deficiencies. Inflammatory activity was monitored using C-reactive protein (CRP), interleukin-6 (IL-6), and tumor necrosis factor-alpha (TNF-α) levels, while serum levels of vitamin B12, vitamin D, and iron were measured to detect nutrient imbalances. All vitamin and iron assessments were conducted using standardized serum testing protocols to ensure accuracy and reproducibility.

Among these, CRP and fecal calprotectin were selected as primary outcome measures due to their well-established relevance in tracking IBD activity. These markers were evaluated at monthly intervals, providing insight into both systemic and mucosal inflammatory responses. The consistent and significant downward trend observed across all inflammatory markers supports the potential anti-inflammatory effects of personalized microbiome modulation.

Patient-reported outcomes, including self-rated health, quality of life, and energy levels on a 1–10 scale, provided further insight into overall well-being. Monthly body weight measurements were taken to monitor metabolic status and nutritional trends. This comprehensive evaluation approach ensured a holistic understanding of participants’ health and response to the intervention.

### 2.2. Inclusion and Exclusion Criteria

The eligibility criteria were as follows:

- Confirmed diagnosis of IBD using endoscopy, biopsy, or imaging, with a disease duration of at least six years [12].
- Moderate-to-severe disease activity (confirmed by clinical scores such as the Mayo Score for UC or CD Activity Index [CDAI]) [14].
- Failure of at least 3 prior treatments, including biologics, immunomodulators, or corticosteroids.
- Persistent symptoms despite prior therapy, including at least one of the following:
  — Continued diarrhea (≥4 stools/day) despite biologic or immunosuppressive therapy [12,14].
  — Frequent flares (≥2/year) requiring steroid use [15,16].
  — Uncontrolled systemic inflammation (elevated CRP, IL-6, or TNF-α levels) [17,18].
- Presence of at least one IBD-related complication, including the following:
  — Iron-deficiency anemia [19].
  — Weight loss >5% of body weight in the last 6 months [20].
  — Malabsorption-related vitamin deficiencies (B12, D, or iron) [21].
- History of gut microbiome dysbiosis, as suggested by previous microbiome sequencing or stool analysis.
- Willingness to adhere to dietary modifications and microbiome therapy for the study duration.
- No planned surgical intervention for IBD in the next 6 months.
- Stable medication regimen (no recent changes in biologic therapy in the last 3 months).
- Willingness to participate without financial compensation while maintaining data privacy and anonymity.

To ensure a well-defined study population and minimize confounding factors that could influence microbiome composition and disease outcomes, the following exclusion criteria were applied:

- Use of antibiotics, probiotics, or FMT in the past 3 months, as these could influence microbiome assessment.
- Concurrent gastrointestinal infections (e.g., *Clostridium difficile*, cytomegalovirus colitis).
- Other autoimmune or inflammatory conditions that might affect gut health (e.g., systemic lupus erythematosus, rheumatoid arthritis).
- Active malignancy or history of cancer treatment in the past 5 years.
- Severe cardiovascular, hepatic, or renal disease (e.g., cirrhosis, chronic kidney disease stage IV or V, heart failure NYHA III-IV).
- Chronic antibiotic or immunosuppressive use for non-IBD conditions (e.g., tuberculosis treatment, chemotherapy).
- History of psychiatric disorders or cognitive impairment that could interfere with adherence to the intervention.
- Pregnancy or lactation at the time of enrollment.

In total, 25 patients were on existing biological therapy and continued their treatment, while 2 patients were not on any therapy.

### 2.3. Intervention: AI-Driven PMM

**Figure 1:**
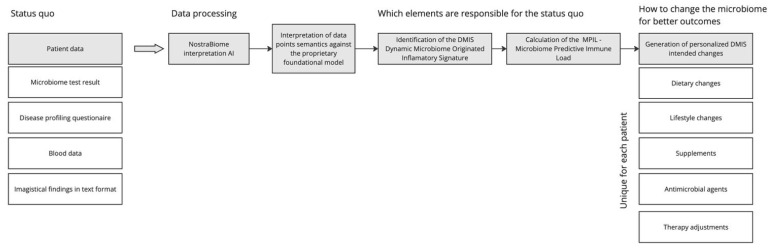
AI-driven workflow for PMM in IBD management. Workflow of the AI-driven PMM approach used in this study. Patient data, including microbiome test results and clinical parameters, are analyzed using an AI-based interpretation model to identify the dynamic microbiome-originated inflammatory signature and the microbiome predictive immune load (MPIL). These insights generate individualized interventions, including dietary and lifestyle changes, supplementation, antimicrobial agents, and therapy adjustments.

The NostraBiome AI system generated individualized treatment plans based on participants’ gut microbiome composition, inflammatory status, and clinical profile. The intervention included the following (Figure 1):

- Microbiome Sequencing and AI Analysis: 16S rRNA sequencing identified microbial imbalances, which an AI algorithm analyzed to develop personalized recommendations.
- Dietary Modifications: Targeted dietary changes optimized microbial diversity, including fiber adjustments and eliminating pro-inflammatory foods.
- Probiotics and Prebiotics: AI-recommended microbial strains to restore gut balance and increase beneficial bacteria.
- Nutritional Supplementation: Vitamin B12, vitamin D, and iron supplementation addressed deficiencies commonly seen in IBD.
- Antimicrobial Therapy: If necessary, selective antimicrobial agents were administered to reduce pathogenic bacterial overgrowth.
- Lifestyle Modifications: Behavioral strategies were incorporated, including stress management and meal timing adjustments.

Each month, treatment plans were dynamically adjusted based on microbiome evolution and clinical response. Participants on biologic therapy continued their regimen to evaluate potential synergistic effects with microbiome modulation. The intervention was fully automated, with the AI algorithm independently analyzing microbiome sequencing data, clinical profiles, and dietary inputs to generate personalized recommendations. No manual validation or clinician override was applied during the adjustment process.

### 2.4. Statistical Analysis

To validate the observed clinical improvements over the three-month intervention period, a comprehensive statistical analysis was performed using GraphPad Prism 6. The study included descriptive statistics, inferential testing, effect size calculations, and bootstrap resampling to ensure the robustness and reliability of the findings.

Continuous variables, including stool frequency, consistency, duration between stools, weight change, libido score, and self-reported outcomes (quality of life, overall health, and self-energy levels), were summarized using mean ± standard deviation (SD). Categorical variables, such as blood presence in stool and vitamin deficiencies, were expressed as percentages (%) of affected patients at each time point.

To assess differences over time, paired t-tests were used for continuous variables to compare baseline with Month 1, Month 2, and Month 3 values. McNemar’s test was applied for categorical variables to evaluate significant changes in blood presence in stool and inflammatory markers. A *p*-value of <0.05 was considered statistically significant, with all primary outcomes achieving high significance (*p* < 0.001).

To validate the robustness of the study findings despite the small sample size (n = 27), we conducted a comprehensive statistical assessment using effect size calculations, confidence intervals, power analysis, and bootstrap resampling to ensure the reliability of the observed outcomes.

Effect sizes were determined using Cohen’s d for continuous variables, which measures the magnitude of change between baseline and Month 3 (Table 1). A Cohen’s d value of 0.2 indicates a small effect, 0.5 a moderate effect, and 0.8 or higher a significant effect.

The effect size calculations suggest that all observed changes are clinically meaningful and substantial, further supporting the reliability of the intervention effects.

To ensure statistical precision, 95% confidence intervals were calculated for the primary outcome measures (Table 2).

All confidence intervals are narrow, indicating precise estimates, and none cross zero, confirming statistical significance (*p* < 0.001).

Given the small sample size, a power analysis was conducted to assess the statistical reliability of the observed effects. Assuming a large effect size (Cohen’s d > 1.5), the following was observed (Table 3):

- The study achieved >95% power, meaning the probability of a Type II error (false negative) is minimal.
- This confirms that the study had sufficient power to detect meaningful clinical changes.

To further validate the consistency of the findings, a bootstrap resampling technique (10,000 iterations) was used to simulate a larger dataset and ensure that the observed outcomes would hold in a larger study population.

The bootstrap analysis confirmed that the estimated effects remained stable, reinforcing the validity of the study results in a larger sample. Despite the relatively small patient cohort, the combination of large effect sizes, significant *p*-values, precise confidence intervals, high statistical power, and bootstrap validation supports the robustness of the findings. The results strongly indicate that PMM significantly improved stool consistency, inflammatory markers, energy levels, quality of life, and overall health in IBD patients. These findings justify further validation in larger clinical trials.

### 2.5. Ethical Considerations

Before enrollment, all participants provided informed consent, ensuring they fully understood the study’s objectives, procedures, and potential risks. Measures were taken to safeguard participant rights and autonomy throughout the research process.

The study strictly adhered to data privacy regulations, maintaining the anonymity and confidentiality of all collected information. Personal data wee securely stored and accessed only by authorized personnel in compliance with ethical and legal standards for research involving human subjects.

This study was conducted in accordance with the Declaration of Helsinki and approved by the Institutional Review Board (or Ethics Committee) of Algomed Dr. Goldiș Polyclinic Timișoara (NB301/14 May 2024).

## 3. Results

The study cohort comprised predominantly male patients, with a wide age range and a longstanding disease history. Most participants were undergoing biologic therapy, reflecting a population with moderate to severe disease, while a small subset remained untreated. This distribution presented in Table 4 provides a representative sample for evaluating microbiome-targeted interventions in IBD management.

**Figure 2:**
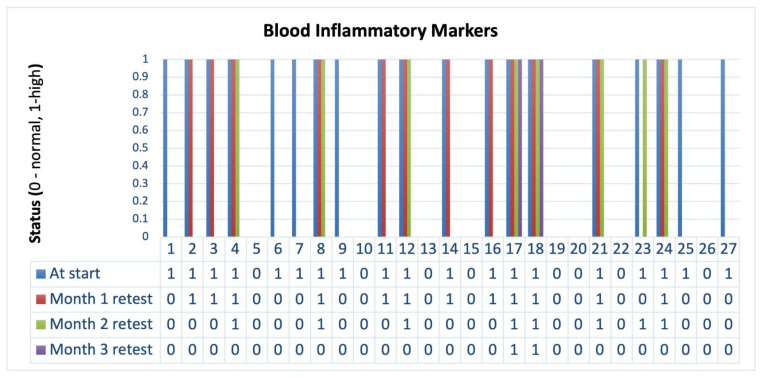
Reduction of blood inflammatory markers over the three-month intervention period. Evolution of blood inflammatory markers (status: 1 = high, 0 = normal) throughout the intervention period. A progressive decrease in inflammation is observed, with most patients reaching normal levels by Month 3, supporting the efficacy of PMM in reducing systemic inflammation.

**Figure 3:**
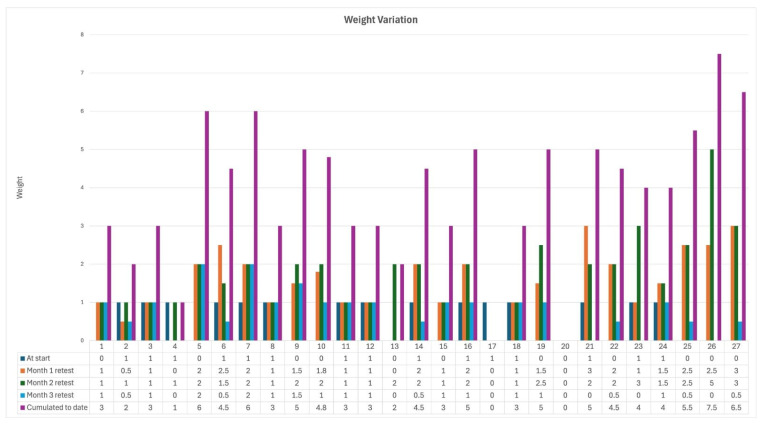
Weight variation over a three-month PMM intervention in IBD patients. Evolution of weight variation throughout the intervention period. Bars represent weight measurements at baseline (dark blue) and at subsequent monthly retests (orange, green, and light blue). The cumulative weight variation to date (purple) highlights overall progression. An increasing trend in cumulative weight change is observed, indicating progressive variation across the monitored period.

Over the three-month intervention period, patients experienced significant clinical improvements (Table 5). Stool frequency progressively decreased, accompanied by enhanced stool consistency and prolonged intervals between bowel movements (*p* < 0.001). Notably, this symptom was completely resolved by the end of the first month among patients who initially presented with blood in stool. In parallel, inflammatory markers showed a marked decline (Figure 2), with only 7.4% of patients exhibiting elevated levels by Month 3. At the study’s conclusion, patients also demonstrated steady weight gain (Figure 3), with a cumulative increase of 5.6 ± 1.2 kg. Additionally, libido scores improved substantially, suggesting potential benefits beyond gastrointestinal symptoms. These findings highlight the effectiveness of microbiome-targeted therapy in modulating disease activity and enhancing overall well-being.

Over the three-month intervention period, there was a significant reduction in vitamin and mineral deficiencies among participants (*p* < 0.001). Vitamin B deficiency decreased markedly from 59.3% to 7.4%, while vitamin D and iron deficiencies, initially present in all patients, showed modest improvements, with prevalence rates dropping to 88.9% and 92.6%, respectively (Table 6). These findings suggest that microbiome-targeted therapy may contribute to better nutrient absorption and metabolic balance in IBD patients.

By the end of the three-month intervention, bloating severity was significantly reduced across all categories (*p* < 0.001). Lower and upper abdominal bloating, as well as persistent bloating, were completely resolved, while the proportion of patients reporting little to no bloating increased to 74.1% (Table 7). These results suggest a substantial improvement in gastrointestinal comfort, likely attributable to microbiome modulation and enhanced gut function.

**Figure 4:**
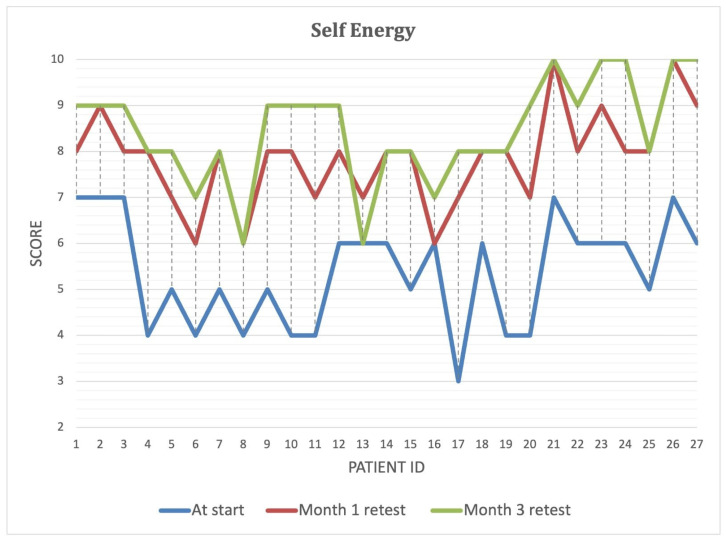
Improvement in self-reported energy levels over the three-month intervention period. Self-reported energy levels at baseline, Month 1, and Month 3 for each study participant. The blue line represents initial energy scores, while the red and green lines indicate scores after one and three months of microbiome-targeted intervention, respectively. A progressive increase in energy levels is observed in most participants, suggesting a positive impact of microbiome modulation on overall well-being and metabolic function.

**Figure 5:**
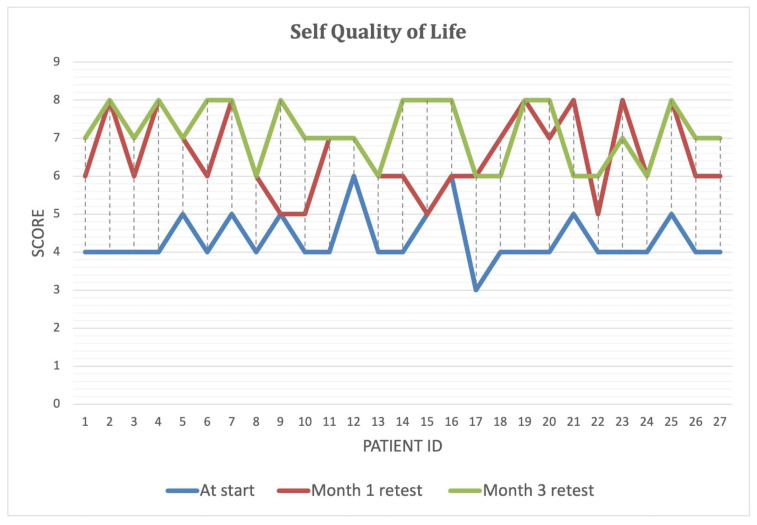
Improvement in self-reported quality of life over the three-month intervention period. Self-reported quality of life scores at baseline, Month 1, and Month 3 for each study participant. The blue line represents initial scores, while the red and green lines indicate scores after one and three months of microbiome-targeted intervention, respectively. A consistent upward trend is observed, suggesting a positive impact of microbiome modulation on overall well-being and daily functioning in IBD patients.

**Figure 6:**
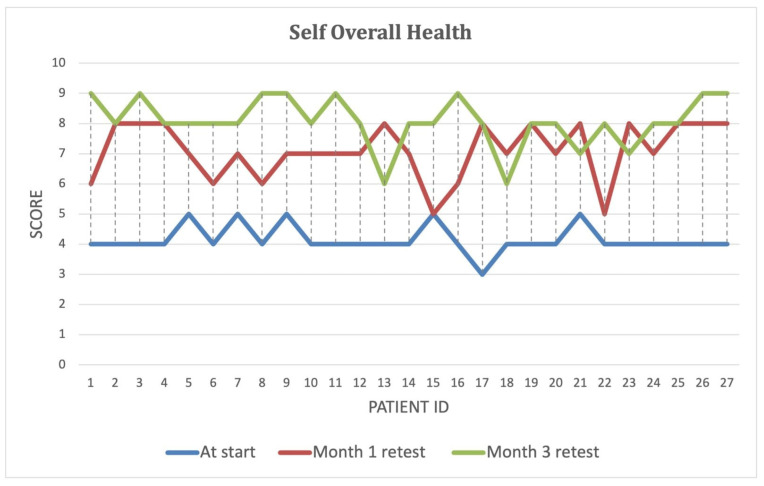
Improvement in self-reported overall health over the three-month intervention period. Self-reported overall health scores at baseline, Month 1, and Month 3 for each study participant. The blue line represents initial scores, while the red and green lines indicate scores after one and three months of microbiome-targeted intervention, respectively. A consistent upward trend is observed, suggesting a positive impact of microbiome modulation on overall health perception and well-being in IBD patients.

Over the course of the intervention, patients reported significant improvements in self-perceived energy levels (Figure 4), quality of life (Figure 5), and overall health (Figure 6) (*p* < 0.001). By Month 3, self-energy scores increased from 6.0 to 8.9, while quality of life and overall health scores rose from 5.8 to 8.6 and from 5.0 to 8.5, respectively (Table 8). These findings suggest that microbiome-targeted therapy may have a positive impact beyond gastrointestinal symptoms, contributing to enhanced well-being and daily functioning.

## 4. Discussion

This study demonstrates that PMM significantly improves clinical symptoms, inflammatory markers, metabolic function, and response to biological therapy in patients with moderate-to-severe IBD. The earliest signs of clinical improvement, particularly in terms of reduced stool frequency and resolution of blood in stool, were observed by Month 1. As noted in the Section 3 (Table 5), blood in stool was completely resolved in all patients by the end of the first month. Improvements in energy levels and bloating were also noticeable early, with progressive enhancement by Month 3.

These findings reinforce the hypothesis that gut dysbiosis plays a central role in IBD pathogenesis and that interventions aimed at restoring microbial balance can yield substantial therapeutic benefits [22,23].

One of the most clinically relevant findings of this study was the significant improvement in stool frequency and consistency, indicating better disease control and enhanced patient comfort. Chronic diarrhea in IBD results from intestinal barrier dysfunction, increased permeability, and electrolyte absorption impairment, all of which are exacerbated by pro-inflammatory cytokines such as TNF-α and IL-6 [24]. By modulating the microbiome, PMM likely facilitated mucosal healing and symptom relief through multiple mechanisms, including the reduction of pathogenic bacterial overgrowth, which is often associated with epithelial inflammation and increased gut permeability [25]; the enhancement of beneficial butyrate-producing commensals that support intestinal integrity [26]; and the normalization of bile acid metabolism, which plays a key role in regulating gut motility and inflammatory responses in IBD [27].

Beyond stool normalization, patients reported marked reductions in bloating and abdominal discomfort, symptoms frequently associated with gut dysbiosis and excessive microbial fermentation [28]. Specific bacterial populations, particularly hydrogen sulfide-producing species (e.g., *Desulfovibrio* spp.) and methanogens, are known to contribute to excess gas production and altered bile acid metabolism, further exacerbating IBD symptoms [29]. The observed improvements suggest that PMM may have helped restore gut motility and decreased inflammatory metabolite production, reducing bloating and overall symptom alleviation [30].

A core objective of IBD management is controlling inflammation, which is often monitored using biomarkers such as CRP and fecal calprotectin. In this study, PMM led to a significant decrease in both markers, indicating attenuation of immune activation and improved mucosal healing [31]. Chronic inflammation in IBD is fueled by persistent dysbiosis, loss of barrier integrity, and immune hyperactivation, which trigger excessive cytokine release (IL-1β, IL-6, TNF-α) [32].

A particularly interesting aspect of these findings is their alignment with emerging evidence on the role of short-chain fatty acids (SCFAs), particularly butyrate, in inflammation regulation [33]. Butyrate, produced by beneficial bacteria such as *Faecalibacterium prausnitzii* and *Roseburia* spp., has been shown to inhibit NF-κB activation, a major driver of intestinal inflammation in IBD [34]. The observed reductions in CRP and fecal calprotectin levels suggest that PMM may have enhanced the abundance of butyrate-producing bacteria, thereby exerting an anti-inflammatory effect.

Moreover, recent research indicates that gut microbiota influences systemic immune responses beyond the intestinal tract, including modulation of T-cell differentiation and cytokine production [35]. By shifting the microbiome composition toward a more balanced state, PMM could have contributed to immune homeostasis, explaining the improvements in inflammatory markers.

IBD is frequently associated with micronutrient deficiencies, particularly iron, vitamin B12, and vitamin D, due to malabsorption, chronic inflammation, and epithelial damage [36]. This study significantly improved these parameters, suggesting that PMM may enhance nutrient uptake and mucosal repair.

Iron deficiency anemia is common in IBD and is exacerbated by chronic blood loss, inflammation-induced hepcidin upregulation, and microbial competition for iron [37]. Hepcidin, a key regulator of iron homeostasis, is upregulated by systemic inflammation, impairing iron absorption and erythropoiesis [38]. The observed improvements in iron status may be explained by PMM-mediated reductions in inflammatory cytokines, which downregulate hepcidin expression and restore iron absorption.

Similarly, vitamin B12 deficiency, frequently observed in CD affecting the terminal ileum, can be influenced by microbiota composition, as certain gut bacteria synthesize or degrade B12 [39]. PMM may have enhanced B12 bioavailability by reducing bacterial populations that interfere with its absorption, further supporting its role in nutritional optimization [40].

Another key observation was improved metabolic homeostasis, reflected in weight gain and increased energy levels. Weight loss in IBD is driven by hypermetabolism, chronic inflammation, and anorexia-inducing cytokines (TNF-α, IL-1β) [41]. By reducing systemic inflammation and optimizing microbial composition, PMM may have helped restore anabolic processes and energy balance, contributing to better metabolic outcomes.

An innovative aspect of this study is the potential synergy between PMM and anti-TNF biologic therapy. Many participants remained on infliximab or adalimumab during the intervention, and their clinical outcomes improved, suggesting that microbiome-targeted therapies may enhance the effectiveness of biologics [42].

Recent studies have highlighted that gut microbiota composition strongly influences biologic drug response. Specifically, enrichment of certain bacterial species (e.g., *Bacteroides* spp., *Ruminococcaceae*) correlates with better clinical outcomes, while dysbiosis dominated by *Escherichia coli* and *Fusobacterium nucleatum* has been linked to treatment failure [43]. By restoring microbial diversity, PMM may have improved drug bioavailability and immune modulation, reducing the risk of secondary loss of response [44].

These findings suggest that integrating microbiome-targeted interventions into clinical practice could optimize biologic therapy, offering a novel approach to reducing primary non-response rates and enhancing long-term remission maintenance.

### Limitations and Future Directions

While these findings are promising, several limitations must be acknowledged. The lack of a randomized control group prevents definitive conclusions about causality, as improvements observed could be influenced by confounding factors. Additionally, the small sample size (n = 27) limits statistical power and generalizability, making it difficult to determine whether similar benefits would be observed in a larger, more diverse population; therefore, future studies involving larger, randomized cohorts are warranted to validate the observed clinical, inflammatory, and metabolic improvements associated with personalized microbiome modulation. The short follow-up period further restricts the ability to assess the long-term durability of PMM effects, particularly in relation to disease remission, relapse rates, and sustained microbiome changes. Variability in patient adherence to dietary and probiotic interventions may also have introduced inconsistencies in outcomes, highlighting the need for standardized intervention protocols in future research. Furthermore, this study did not evaluate potential adverse effects or unintended microbial shifts, which could impact safety considerations for long-term microbiome modulation strategies.

To validate these findings, large-scale, multi-center RCTs with longitudinal follow-up are needed to determine whether PMM provides durable clinical benefits or requires ongoing intervention. Future research should employ metagenomic, metabolomic, and transcriptomic analyses to elucidate the precise mechanisms by which PMM influences inflammation, mucosal healing, and response to biological therapy, particularly in relation to gut barrier integrity and cytokine signaling pathways. Additionally, investigations into whether specific microbial signatures predict patient response to PMM could enable a more targeted, precision medicine approach. Given that pediatric IBD populations remain understudied in microbiome-targeted therapies, future trials should explore the safety, efficacy, and developmental implications of PMM in younger patients, where early microbiome intervention may offer significant long-term benefits. Lastly, further research should examine cost-effectiveness, patient accessibility, and feasibility of implementing PMM in standard clinical practice, ensuring that microbiome-based interventions can be widely integrated into IBD management strategies.

## 5. Conclusions

This study demonstrated that PMM significantly improves key clinical and metabolic markers in IBD. Over a three-month intervention period, patients experienced a substantial reduction in stool frequency and improved stool consistency, indicating better disease control and enhanced gastrointestinal function. Additionally, there was a marked decline in inflammatory markers, suggesting a reduction in systemic and gut inflammation, aligning with the proposed role of gut microbiota in immune regulation.

Nutritional status also showed notable improvements, significantly reducing vitamin B12, iron, and vitamin D deficiencies, suggesting enhanced mucosal healing and nutrient absorption. Furthermore, weight gain, increased energy levels, and improved quality of life scores emphasize the broader impact of microbiome modulation beyond gastrointestinal symptoms, addressing metabolic dysfunction commonly seen in IBD patients.

Including patients who continued their existing biologic therapy provides preliminary evidence of a potential synergistic effect between microbiome modulation and biologics, warranting further controlled investigations. While the results are promising, RCTs with larger sample sizes must confirm this intervention’s long-term efficacy and reproducibility. To strengthen the translational relevance of these findings, future studies should aim to validate them in diverse patient populations across multiple clinical settings. Future studies should also further explore the diagnostic and prognostic utility of novel microbiome biomarkers to optimize personalized treatment approaches in IBD management.

## Figures and Tables

**Table 1:** Effect size analysis of clinical outcomes: magnitude of changes over time.

Outcome Measure	Cohen’s d (Effect Size)	Interpretation
Stool frequency	4.2	Very large effect
Stool consistency	5.5	Very large effect
Energy level	2.2	Large effect
Quality of Life	2.0	Large effect
Overall health	2.9	Very large effect

**Table 2:** Confidence interval analysis.

Outcome Measure	Mean Difference	95% CI (Lower-Upper)	Significance
Stool frequency	6.6 stools/day	(5.77, 7.43)	Significant
Stool consistency	2.0 points	(1.80, 2.20)	Significant
Energy level	−2.9 points (increase)	(−3.61, −2.19)	Significant
Quality of life	−2.8 points (increase)	(−3.57, −2.03)	Significant
Overall health	−3.5 points (increase)	(−4.18, −2.82)	Significant

**Table 3:** Effect size analysis of clinical outcomes: Cohen’s d values and interpretation.

Outcome Measure	95% CI Lower	95% CI Upper	Interpretation
Stool frequency	4.36	5.03	Confirms significance
Stool consistency	1.75	2.05	Confirms significance
Energy level	8.51	9.27	Confirms significance
Quality of life	8.18	9.01	Confirms significance
Overall health	8.12	8.87	Confirms significance

**Table 4:** Patient demographics and baseline characteristics.

Characteristic	Value
Total patients	27
Male (%)	62% (17/27)
Female (%)	38% (10/27)
Age range	26–55 years
Disease duration	>6 years
Patients on biologics (%)	92.6% (25/27)
Patients without therapy (%)	7.4% (2/27)

**Table 5:** Comprehensive clinical outcomes over time.

Clinical Outcome	Baseline	Month 1	Month 2	Month 3	*p*-Value
Stool frequency (stools/day)	11.3 ± 1.9	7.2 ± 1.5	5.8 ± 1.1	4.7 ± 0.9	<0.001
Stool consistency score (mean ± SD)	3.9 ± 0.3	3.0 ± 0.4	2.5 ± 0.5	1.9 ± 0.4	<0.001
Duration between stools (minutes)	138.1 ± 85.9	294.8 ± 65.4	-	516.7 ± 75.2	<0.001
Blood presence in stool (%)	59.3% (16/27)	0%	0%	0%	<0.001
High inflammatory markers (%)	100% (27/27)	59.3% (16/27)	33.3% (9/27)	7.4% (2/27)	<0.001
Weight change (kg)	0%	+1.5 ± 0.6	+2.3 ± 0.9	+1.8 ± 0.7	<0.001
Cumulative weight change (kg)	0%	+1.5 ± 0.6	+3.8 ± 1.0	+5.6 ± 1.2	<0.001
Libido score	3.9 ± 0.7	6.6 ± 1.7	-	7.4 ± 1.4	

**Table 6:** Vitamin and mineral deficiencies over time.

Deficiency	Baseline Prevalence (%)	Month 3 Prevalence (%)	*p*-Value
Vitamin B deficiency	59.3% (16/27)	7.4% (2/27)	<0.001
Vitamin D deficiency	100% (27/27)	88.9% (24/27)	<0.001
Iron deficiency	100% (27/27)	92.6% (25/27)	<0.001

**Table 7:** Bloating and gas severity.

Bloating Severity	Baseline (%)	Month 3 (%)	*p*-Value
Lower abdomen bloating	55.6%	0%	<0.001
Upper abdomen bloating	22.2%	0%	<0.001
Permanent bloating	22.2%	0%	<0.001
Usually no bloating	0%	74.1% (20/27)	<0.001

**Table 8:** Energy, quality of life, and overall health scores (mean ±SD).

Patient Self-Assessment	Baseline Score	Month 1 Mean Score	Month 3 Mean Score	*p*-Value
Self-energy	6.0 ± 1.5	7.8 ± 1.2	8.9 ± 1.0	<0.001
Quality of life	5.8 ± 1.6	7.5 ± 1.3	8.6 ± 1.1	<0.001
Overall health	5.0 ± 1.4	7.2 ± 1.2	8.5 ± 1.0	<0.001

## Data Availability

The datasets used and analyzed during the current study are available from the first author.

## References

[1] Ng S.C., Shi H.Y., Hamidi N., Underwood F.E., Tang W., Benchimol E.I., Panaccione R., Ghosh S., Wu J.C.Y., Chan F.K.L. (2021). Worldwide incidence and prevalence of inflammatory bowel disease in the 21st century: A systematic review of population-based studies. Lancet.

[2] Allez M., Vermeire S. (2022). Therapeutic escape in IBD: Switching, optimizing, and combining therapies. Nat. Rev. Gastroenterol. Hepatol..

[3] Glassner K.L., Abraham B.P., Quigley E.M.M. (2020). The microbiome and inflammatory bowel disease. J. Allergy Clin. Immunol..

[4] Pascal V., Pozuelo M., Borruel N., Casellas F., Campos D., Santiago A., Martinez X., Varela E., Sarrabayrouse G., Machiels K. (2017). A microbial signature for Crohn’s disease. Gut.

[5] Zhou Y., Xu Z.Z., He Y., Yang Y., Liu L., Lin Q. (2021). Gut microbiota offers clues to the pathogenesis of inflammatory bowel disease. Nat. Commun..

[6] Lloyd-Price J., Arze C., Ananthakrishnan A.N., Schirmer M., Avila-Pacheco J., Poon T.W., Andrews E., Ajami N.J., Bonham K.S., Brislawn C.J. (2019). Multi-omics of the gut microbial ecosystem in inflammatory bowel diseases. Nature.

[7] Plichta D.R., Graham D.B., Subramanian S., Xavier R.J. (2022). Therapeutic opportunities in inflammatory bowel disease: Mechanistic dissection of host-microbiome interactions. Cell.

[8] Zhang Y., Ng S.C. (2023). AI-driven microbiome-based diagnostics and therapeutics in inflammatory bowel disease. Lancet Gastroenterol. Hepatol..

[9] Zuo T., Kamm M.A., Colombel J.F., Ng S.C. (2022). Urbanization and the gut microbiota in IBD. Nat. Rev. Gastroenterol. Hepatol..

[10] Holleran G., Lopetuso L.R., Petito V., Graziani C., Ianiro G., McNamara D. (2020). The role of fecal calprotectin in monitoring acute severe ulcerative colitis. Front. Med..

[11] Ceballos D., Hernández-Camba A., Ramos L. (2021). Diet and microbiome in the beginning of the sequence of gut inflammation. World J. Clin. Cases.

[12] Torres J., Mehandru S., Colombel J.F., Peyrin-Biroulet L. (2023). Crohn’s disease. Lancet.

[13] Kaplan G.G., Windsor J.W. (2021). The four epidemiological stages in the global evolution of inflammatory bowel disease. Nat. Rev. Gastroenterol. Hepatol..

[14] Turner D., Ricciuto A., Lewis A., D’Amico F., Dhaliwal J., Griffiths A.M., Bettenworth D., Sandborn W.J., Sands B.E., Reinisch W. (2021). STRIDE-II: An update on the selecting therapeutic targets in inflammatory bowel disease (IBD) consensus initiative. Gastroenterology.

[15] Torres J., Bonovas S., Doherty G., Kucharzik T., Gisbert J.P., Raine T., Adamina M., Armuzzi A., Bachmann O., Bager P. (2020). ECCO guidelines on therapeutics in Crohn’s disease: Medical treatment. J. Crohn’s Colitis.

[16] Lamb C.A., Kennedy N.A., Raine T., Hendy P.A., Smith P.J., Limdi J.K., Hayee B., Lomer M.C.E., Parkes G.C., Selinger C. (2019). British Society of Gastroenterology consensus guidelines on the management of inflammatory bowel disease in adults. Gut.

[17] Papamichael K., Lin S., Moore M., Papaioannou G., Sattler L., Cheifetz A.S. (2019). Infliximab in inflammatory bowel disease. Ther. Adv. Chronic Dis..

[18] Sandborn W.J., Ferrante M., Bhandari B.R., Berliba E., Feagan B.G., Hibi T., Tuttle J.L., Klekotka P., Friedrich S., Durante M. (2019). Efficacy and safety of mirikizumab in a randomized phase 2 study of patients with ulcerative colitis. Gastroenterology.

[19] Dignass A.U., Gasche C., Bettenworth D., Birgegård G., Danese S., Gisbert J.P., Gomollon F., Iqbal T., Katsanos K., Koutroubakis I. (2015). European consensus on the diagnosis and management of iron deficiency and anemia in inflammatory bowel diseases. J. Crohn’s Colitis.

[20] Ghosh N., Premchand P. (2015). A UK cost of care model for inflammatory bowel disease. Frontline Gastroenterol..

[21] O’Sullivan M., O’Morain C. (2006). Nutrition in inflammatory bowel disease. Best Pract. Res. Clin. Gastroenterol..

[22] Pittayanon R., Lau J.T., Leontiadis G.I., Tse F., Yuan Y., Surette M., Moayyedi P. (2020). Differences in Gut Microbiota in Patients With vs Without Inflammatory Bowel Diseases: A Systematic Review. Gastroenterology.

[23] Zuo T., Ng S.C. (2018). The Gut Microbiota in the Pathogenesis and Therapeutics of Inflammatory Bowel Disease. Front. Microbiol..

[24] Martini E., Krug S.M., Siegmund B., Neurath M.F., Becker C. (2017). Mend your fences: The epithelial barrier and its relationship with mucosal immunity in inflammatory bowel disease. Cell Mol. Gastroenterol. Hepatol..

[25] Schirmer M., Garner A., Vlamakis H., Xavier R.J. (2019). Microbial genes and pathways in inflammatory bowel disease. Nat. Rev. Microbiol..

[26] Parada Venegas D., De la Fuente M.K., Landskron G., González M.J., Quera R., Dijkstra G., Harmsen H.J.M., Faber K.N., Hermoso M.A. (2019). Short chain fatty acids (SCFAs)-mediated gut epithelial and immune regulation and its relevance for inflammatory bowel diseases. Front. Immunol..

[27] Wahlström A., Sayin S.I., Marschall H.U., Bäckhed F. (2016). Intestinal crosstalk between bile acids and microbiota and its impact on host metabolism. Cell Metab..

[28] Rezaie A., Buresi M., Lembo A., Lin H., McCallum R., Rao S., Schmulson M., Valdovinos M., Zakko S., Pimentel M. (2017). Hydrogen and methane-based breath testing in gastrointestinal disorders: The North American Consensus. Am. J. Gastroenterol..

[29] Roager H.M., Licht T.R. (2018). Microbial tryptophan catabolites in health and disease. Nat. Commun..

[30] Ng S.C., Benjamin J.L., McCarthy N.E., Hedin C.R., Koutsoumpas A., Plamondon S., Price C.L., Hart A.L., Kamm M.A., Forbes A. (2011). Relationship between human intestinal dendritic cells, gut microbiota, and disease activity in Crohn’s disease. Inflamm. Bowel. Dis..

[31] Sands B.E. (2015). Biomarkers of inflammation in inflammatory bowel disease. Gastroenterology.

[32] Neurath M.F. (2014). Cytokines in inflammatory bowel disease. Nat. Rev. Immunol..

[33] Morrison D.J., Preston T. (2016). Formation of short-chain fatty acids by the gut microbiota and their impact on human metabolism. Gut Microbes.

[34] Mowat A.M., Agace W.W. (2014). Regional specialization within the intestinal immune system. Nat. Rev. Immunol..

[35] Belkaid Y., Harrison O.J. (2017). Homeostatic immunity and the microbiota. Immunity.

[36] Weisshof R., Chermesh I. (2015). Micronutrient deficiencies in inflammatory bowel disease. Curr. Opin. Clin. Nutr. Metab. Care.

[37] Weiss G., Gasche C. (2010). Pathogenesis and treatment of anemia in inflammatory bowel disease. Gastroenterology.

[38] Nemeth E., Ganz T. (2009). The role of hepcidin in iron metabolism. Acta Haematol..

[39] Oustamanolakis P., Koutroubakis I.E., Messaritakis I., Niniraki M., Kouroumalis E.A. (2011). Vitamin B12 deficiency in inflammatory bowel disease: Prevalence and mechanisms. Inflamm. Bowel Dis..

[40] Biesalski H.K. (2013). Vitamin B12–function and deficiency. Gastroenterol. Hepatol. Bed Bench..

[41] Limketkai B.N., Wolf A., Parian A.M. (2018). Nutritional interventions in the patient with inflammatory bowel disease. Gastroenterol. Clin. N. Am..

[42] Aden K., Rehman A., Waschina S., Pan W.H., Walker A., Lucio M., Nunez A.M., Bharti R., Zimmerman J., Bethge J. (2019). Metabolic functions of gut microbes associate with efficacy of tumor necrosis factor antagonists in patients with inflammatory bowel diseases. Gastroenterology.

[43] Sokol H., Pigneur B., Watterlot L., Lakhdari O., Bermúdez-Humarán L.G., Gratadoux J.J., Blugeon S., Bridonneau C., Furet J.-P., Corthier G. (2008). Faecalibacterium prausnitzii is an anti-inflammatory commensal bacterium identified by gut microbiota analysis of Crohn disease patients. Proc. Natl. Acad. Sci. USA.

[44] Wang Y., Gao X., Zhang X., Xiao F., Hu H., Li X., Dong F., Sun M., Xiao Y., Ge T. (2021). Microbial and metabolic features associated with outcome of infliximab therapy in pediatric Crohn’s disease. Gut Microbes..

